# Localized surface plasmon resonance sensing of Trenbolone acetate dopant using silver nanoparticles

**DOI:** 10.1038/s41598-024-56456-w

**Published:** 2024-03-08

**Authors:** Moses Wabwile Juma, Zephania Birech, Nancy Mwikali Mwenze, Annah Moraa Ondieki, Malik Maaza, Simon Dhlamini Mokhotjwa

**Affiliations:** 1 UNESCO-UNISA Africa Chair in Nanoscience and Nanotechnology (U2ACN2), College of Graduate Studies, University of South Africa (UNISA), Pretoria, South Africa; 2 NANOAFNET, iThemba LABS-National Research Foundation of South Africa, 1 Old Faure Road, Cape Town, 7129 Western Cape South Africa; 3 Department of Physics, University of South Africa, Muckleneuk Ridge, Pretoria, 0001 South Africa; 4 Laser Physics and Spectroscopy Research Group, Department of Physics, University of Nairobi, P.O Box 30197-00100, Nairobi, Kenya

**Keywords:** Trenbolone acetate, Doping, Localized surface plasmon resonance (LSPR), Density functional theory (DFT), Nanoscale biophysics, Biosensors

## Abstract

In this work, localized surface plasmon resonance (LSPR) sensing as applicable in the detection of Trenbolone acetate dopant is demonstrated. We show that the LSPR of the Trenbolone acetate/silver nanoparticle (Tren Ac/AgNPs) complex is sensitive to changes in the adsorbent concentration. The results show an average redshift of + 18 nm in the LSPR peak with variations in intensity and broadening behavior of the LSPR band of the Tren Ac/AgNPs complex. AgNPs were synthesized using laser ablation in liquid (LAL) technique with water as the solvent. UV–Vis spectroscopy was used for absorbance measurements and particle size and morphology were monitored using scanning electron microscopy (SEM). The aggregation behavior of the Tren Ac/AgNPs complex was monitored using energy-dispersive X-ray spectroscopy (EDS). Molecular Electrostatic Potential (MEP) and the HOMO–LUMO orbitals of the optimized Trenbolone acetate structure were obtained using Density Function Theory (DFT). The molecule was optimized at the B3LYP level of theory using the 6–311 basis set carried out using the Gaussian 09 software package. The results showed that O^2−^ is Trenbolone acetate’s active site that would interact with Ag^+^ to form a complex that would influence the plasmon behavior. The results presented in this work demonstrate the feasibility of LSPR for anabolic androgenic steroid detection.

## Introduction

Surface plasmon resonance-based sensors have received wide applications as they are sensitive, specific, quantitative, and label-free^1^. Their sensing is based on the fact that the adsorption of molecules on the nanoparticle/ metal surface results in changes in the localized surface plasmon resonance/surface plasmon resonance (LSPR/SPR) in terms of wavelength shifts and intensity changes. Such high sensitivity has resulted in the development of biosensors^2^. The angle that triggers the SPR/LSPR is often dependent on the refractive index of the material near the surface^3^, such that any changes in the refractive index form the basis for analyte detection even at low detection levels^4^.

Biosensing has benefited from the signal amplification behavior of nanoparticles. For instance,^5^ used iron oxide nanoparticles to develop a sensor that achieves an ultrasensitive detection of prion disease that is associated with isoform. The strong electromagnetic fields generated around metallic nanoparticles during surface plasmon resonance can enhance the local electromagnetic field intensity. This enhancement leads to increased sensitivity in detecting analytes through techniques such as surface-enhanced Raman spectroscopy (SERS)^6^. Compared to bulk materials, metallic nanoparticles have a high surface area to volume ratio that provides ample sites for the attachment of target molecules or biomarkers, maximizing the interaction between the analyte and the sensing surface, thereby amplifying the signals^7^. The other advantage of metallic nanoparticles that makes them suitable for signal enhancement is the plasmonic resonance frequency that can be tuned by controlling the particle size, shape, composition, and surrounding environment. This tunability enables optimization of the nanoparticles' optical response for specific applications, further enhancing signal amplification^8^.

The advantages of metal surfaces are enhanced when the metals are separated into particles whose wavelengths are smaller than the wavelength of light^9,10^. The light incident on the nanoparticles introduces conduction electrons in the particles making them collectively resonate with a frequency that is dependent on the size, shape, and nature of the nanoparticles^11^. The LSPR modes make the nanoparticles scatter light intensely with a wide scattering cross-section. The phenomenon allows nanomaterials to be used for surface enhancement, biochemical sensor applications^12^, and intense labels for immunoassays^13^. The advantage of metallic nanomaterials is that they can be synthesized and modified with different chemical functional groups making it possible for them to be conjugated with drugs for diverse analytical purposes^14^. Similarly, metallic nanoparticles allow bio-specific interactions that aid in bio-detection based on the localized surface plasmon resonance band modification. The modification includes changing the spectral characteristics of the local environment of the metallic nanomaterials^15^.

Silver nanoparticles, in particular, have found greater utility in biosensing applications. Beck et al.^16^ notes that the unique physical, electrochemical, and optical properties of silver nanoparticles make them suitable for biosensing and point-of-care applications. The inherent capabilities of silver nanoparticles improve their sensor performance hence enabling them to attain analyte detection at low concentrations with a low sample volume demand^16^. According to^17^, the various optical properties make silver nanoparticles an effective metal for making transducers used in biosensing. Silver nanoparticles were used in this work since they have freely moving electrons which when excited by high-energy photons result in a surface plasmon resonance and a subsequent strong absorption that is dependent on the shape, size, and dispersion^18^. When compared to other nanoparticles, silver is superior since it has a higher molar extinction coefficient and a narrow SPR band, especially in the visible region^8^. As the distance between particles decreases, the plasmonic fields of each particle start to overlap, resulting in a shift towards higher wavelengths or a reduction in absorption^8^. Therefore, the chemistry of the interaction between nanoparticles and target analytes presents opportunities for the detection of analytes at the sub-ppb levels^19^.

Trenbolone is an anabolic steroid that improves workout output by enhancing muscles. Although the molecule has found extensive use in livestock due to its ability to build animal mass, it has been classified as an anabolic androgenic steroid that creates an unfair competitive advantage in sports^20^. WADA often uses analytic, science-based approaches such as tandem mass spectrometry (MS) and gas or liquid chromatography to detect doping. However, the use of nanoparticles has illustrated significant potential in biomolecular-sensing applications and its introduction to anti-doping detection is of great importance^21^. Furthermore, illicit recreational drugs, substances that can be potentially abused, and drugs with dosage limitations according to the prohibited lists announced by the World Antidoping Agency (WADA) are becoming of increasing interest to forensic chemists. The main emphasis is on the advantages that noble metal nanoparticles bring to optical biosensors for signal enhancement and the development of highly sensitive (label-free) biosensors. Soon, such optical biosensors may be an invaluable substitute for conventional anti-doping detection methods such as chromatography-based approaches, and may even be commercialized for routine anti-doping tests^21,22^.

Using nanoparticles provides a new frontier with remarkable potential in doping campaigns. Izquierdo-Lorenzo et al.^23^ demonstrated that it’s possible to detect dopants on metallic nanoparticles by exploring the adsorption mechanism. Izquierdo-Lorenzo et al. ^23^ combined surface-enhanced Raman spectroscopy and plasmon resonance for ultrasensitive detection of aminoglutethimide drug. Malekzad et al.^21^ explained that one of the benefits of metal nanoparticles in optical biosensing is the ability to enhance signals and being a highly sensitive/label-free approach. Consequently, optical biosensors may in the future, be a significant substitute for the conventional anti-doping detection approaches.

The growth of plasmon resonance both in anti-doping campaigns and in any analyte detection, is of great interest. Although both^21,23^ highlight plasmon resonance as a great contributor to analyte/dopant detection, little effort has gone into providing an in-depth understanding of the interactive behavior of nanoparticles (silver in particular) and any dopant/analyte. For the field of nano-based sensing to grow especially in the anti-doping campaign, a detailed impact of analytes on the plasmon behavior of nanoparticles is key^24^. For instance, Gutiérrez-Gallego et al.^25^ noted that using surface plasmon resonance harbors a promise for the direct fight against doping, both for gene doping, steroid, and protein hormone doping. Consequently, this work contributes to dopant detection by understanding the science of plasmon resonance of Trenbolone Acetate when mixed with silver nanoparticles as a nano signal enhancer. The work is also based on the premise that the intensity and band position of the LSPR depends on several factors including the dielectric properties of the environment^10,26,27^. The changes allow for optical sensors to be used in the detection of analytes in biomedical applications.

## Experimental methods

### Reagents and Instrumentation

Analytical grade silver granules (99.99% purity) were obtained from Sigma Aldrich while Ultimate Precision Anabolic (UPA) Trenbolone acetate was purchased from Anabolic Alot (South Africa). Absorbance measurements were carried out using two sets of spectrometers. A UV–Vis Spectrophotometer (solid spec-3700 DUV230, A11094500005) in the range of 250–800 nm at room temperature (at Physics Department, University of Nairobi) when evaluating the Lambert–Beer law and Ocean Optics units within the spectral range of interest of 250–800 nm at Ithemba LABS South Africa for the second set of ppb Trenbolone Ac concentrations. In both cases, stock solutions were made before serial diluting to provide lower concentrations. Particle sizes were explored using a NOVA NANOSEM 230 scanning electron microscope based at the Electron Microscope Unit; at the University of Capetown.

### Synthesis of silver nanoparticles

Silver nanoparticles were synthesized using laser ablation in liquid (LAL) technique with water as the solvent. A 1064 nm laser with a Q-switch delay was used to ablate silver granules and generate nanoparticles. The laser energy was maintained at 250 mJ, Q-switch delay of 2 ns, and pulse repetition frequency of 5 Hz while the ablation time was kept at eight hours after optimization as described by^28^. A detailed explanation of the laser ablation in liquid approach as used in this work was previously reported by^29^.

## Results and discussion

### Evaluating the concentration limit for the Beer–Lambert law

**Figure 1:**
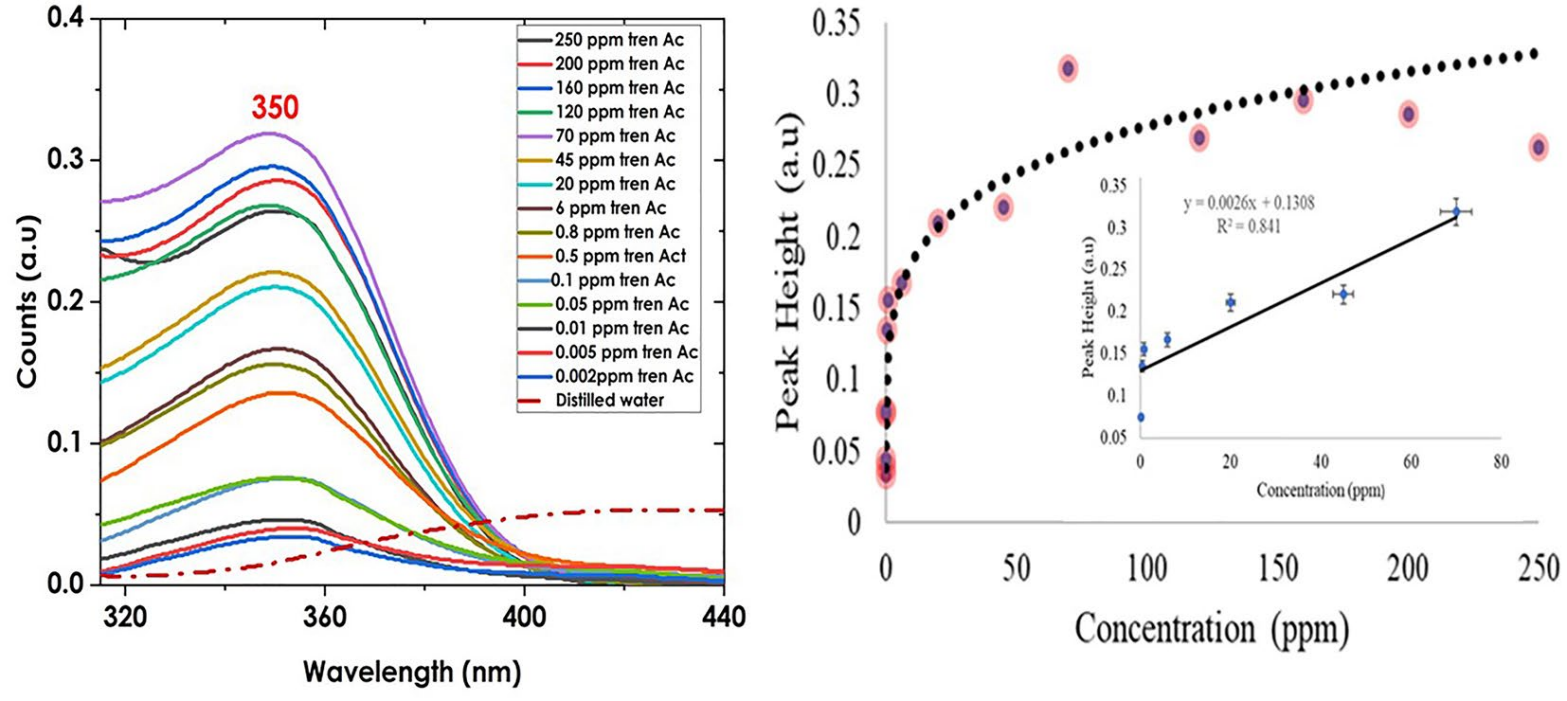
UV–Vis spectra of Trenbolone showing the absorbance band at 349 nm (left) and the effect of concentration to signal intensity (right; the inset show the calibration for concentrations between 0.1 and 70 ppm) depicting the limit of Lambert Beer Law.

There is little work done to characterize Trenbolone using UV–Vis and thus, to the best of our knowledge, this will be pioneer research in this area. Figure 1 shows the absorbance spectra of Trenbolone (in distilled water) at different concentrations. It is understood, according to Beer–Lambert law that at low concentrations, absorption is proportional to the concentration of the solute^30^. Therefore, at such concentrations, the more the number of particles within a solution, the higher the absorbance value since each particle will absorb a significant amount of light^31^.

UV–Vis is often based on the selective absorption of electromagnetic radiation within a given wavelength range. For Trenbolone diluted in distilled water, absorption occurred at around 349 nm. The data background was subtracted to remove background artifacts and the spectral wavelength range was maintained between 310 and 440 nm to clearly show the absorbance band position.

One notable observation is the linear trend observed in the absorbance as the concentration of trenbolone increases at ≤ 70 ppm. This result is expected since in spectroscopy, two critical factors that explain absorbance are the concentration of the absorbing molecules and the path length as explained using Beer–Lambert law;

1 $${\text{A}} = \varepsilon *{\text{b}}*{\text{C}}$$

where $A$ is absorbance, ε is the coefficient of molar exclusion, b is the cuvette's thickness and C is the analyte's concentration. From this equation, it is expected that concentration linearly correlates with absorbance since at higher concentrations, more particles absorb light that enters hence increasing the absorbance.

The Beer–Lambert law fails to be observed beyond 70 ppm. One of the reasons for the failure in the linear trend especially beyond 70 ppm is increased coulomb interactions as molecules are likely to interact with each other affecting the electronic structure of the absorbing species^32^. At high concentrations, the molecules of the substance will likely interact with one another hence altering the molar absorptivity coefficient that plays a key role in finding absorbance^33^. Mayerhöfer et al.^33^ also suggest that at higher concentrations, light tends to travel extra distances inside matter (as a result of more particles) due to the scattering and absorbance effects of the material. Therefore, the output intensity at such high concentrations tends to be more attenuated. According to^34^, when light passes through a mixture, attenuation of the light intensity can occur by two mechanisms; for a homogeneous and single-phase solution, the absorbance contributes to attenuation. However, for suspensions having mixtures that have two or more phases (like the case in this research), there is light scattering as a result of differences in refractive indices of AgNPs and Trenbolone^35^. As the concentration increases, light scattering due to the changes in the refractive index of the two species becomes more pronounced, hence the possible failure of the Beer–Lambert law at higher concentrations. According to^36^, at such concentrations where the law fails, the absorbed radiations tend to be absorbed back by self-quenching which is manifested as a decrease in absorption intensity and a reduced nanoparticle activity. The mechanism of self-quenching can also result in the formation of a shell around the nanoparticles hence altering the sensing behavior^37–39^. Another possible reason for the failure of Beer Lambert law is the luminescence behavior of trenbolone acetate which makes the fluorescent emission reach the detector thus reducing the absorbance signal at higher concentrations^40^. The fluorescence emission may as well be re-absorbed by the sample causing further deviation to the Beer Lambert law.

**Figure 2:**
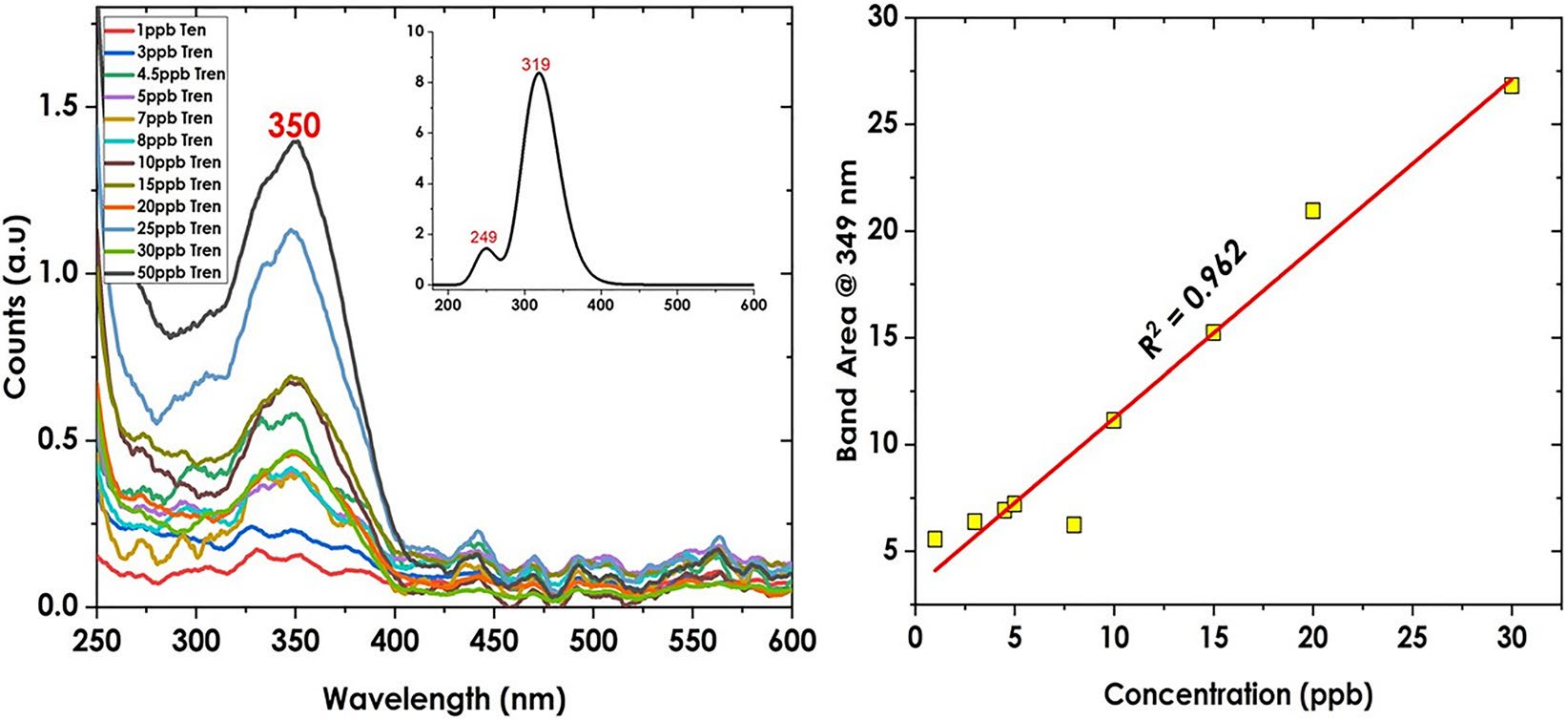
UV–VIS spectra of trenbolone for the biosensing region of interest (left) and on the calibration curve (right).

To ensure that the biosensing technique works within the reported detection limits approved by WADA, a different set of trenbolone concentrations was made with a focus on the lower concentrations that are within the trenbolone detection limits. A set of 12 new samples were prepared and their UV–Vis spectra are shown in Fig. 2.

The lowest detectable amount of Trenbolone and other anabolic steroids depends on, among other factors, the analytical technique and the sample matrix^41^. For instance, in human urine, WADA’s sensitivity requirement for dopants is set to 2–10 ppb. Using the Gas–liquid chromatography technique and urine sample matrix, Putz et al.^42^ and Brun et al.^43^ stated that the average detection limit of trenbolone is 4 ppb. The low detection limit demonstrates the need for high-sensitivity techniques to conform to WADA standards. Therefore, UV–Vis spectroscopy was employed to try and characterize Trenbolone at low concentration levels between 1 and 50 ppb which falls within the detection limit reported in literature. The area was explored using the integrate function in origin 2021, version 9.8,0.2001. The calibration curve for the analyte concentration within this range is given in Fig. 2 (right side).

**Figure 3:**
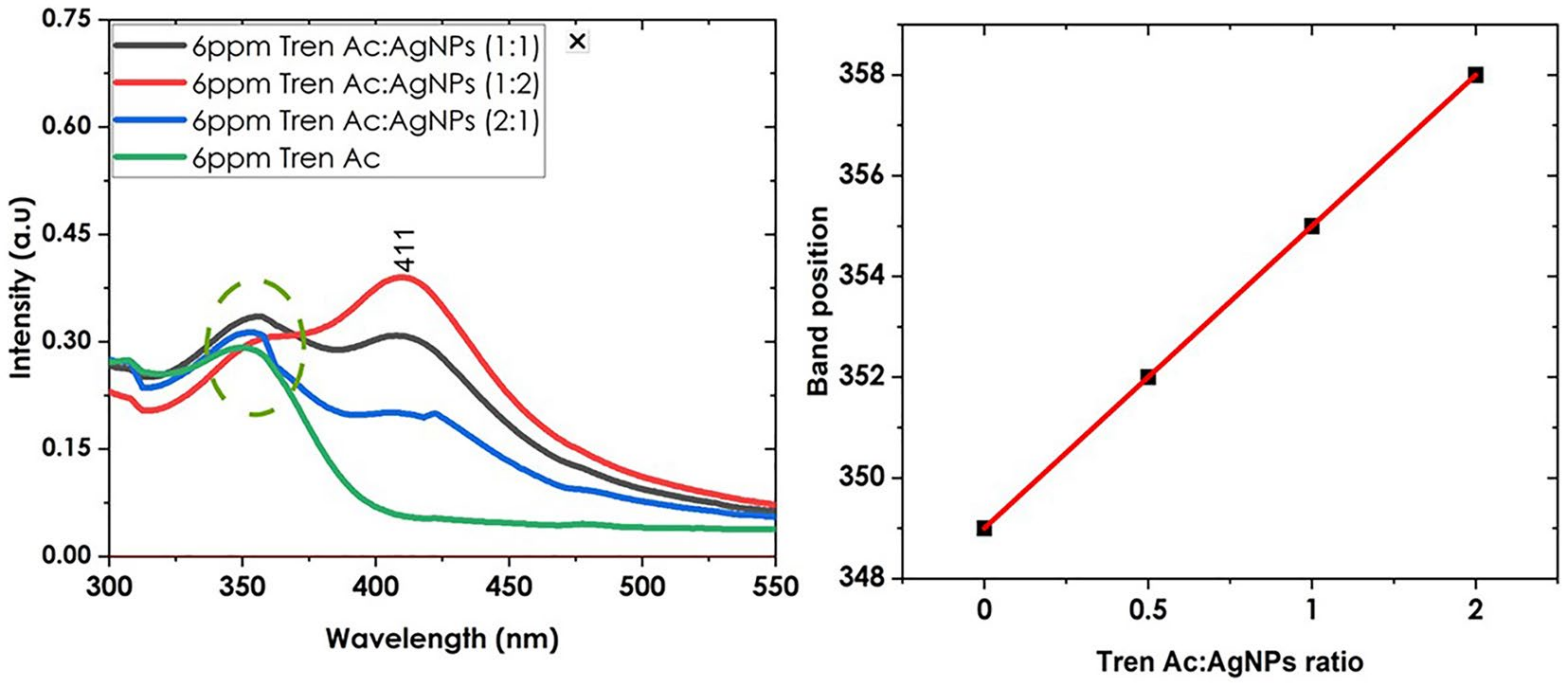
Ratio of Tren Ac to Silver nanoparticles (left) and the influence of AgNPs concentration on the Tren Ac band position (right).

In Fig. 3, Beer Lambert law is inspected providing a linear correlation with an R^2^ value of 96.2%. The limit of detection (LOD) based on the sensitivity curve is obtained as:

2 $$LOD=\frac{3\sigma }{S} x$$

where σ is given as √n * standard error of the y-intercept of the regression line and n is the number of variables. S is the slope of the regression line. Using the formula, the LOD was determined to be 9.12 ppb which falls within the recommended WADA limit. After assessing the absorbance band position of Trenbolone Acetate, the next step was to assess the band when mixed with silver nanoparticles. However, before this step was made, it was first important to optimize the ratio in which silver nanoparticles are to be mixed with the analyte.

### Optimizing the Trenbolone: AgNPs ratio when using AgNPs in biosensing

The volume ratio of AgNPs to the target analyte has a significant impact on the detection limit for UV–Vis spectroscopy^44^. Therefore, the ratio of Tren Ac to silver nanoparticles was explored before subsequent characterizations were carried out. Figure 3 indicates the impact of varying the volume of the silver nanoparticles on the LSPR band for silver nanoparticles and the absorbance band of Trenbolone. The ratio of trenbolone to silver nanoparticles influenced both the absorbance band position of Trenbolone and the localized surface plasmon resonance band of silver nanoparticles.

For Trenbolone, there is a slight red shift in the absorbance with somewhat bigger shifts being recorded for higher silver nanoparticle volumes (see the red spectra in Fig. 3; left). As noted in this spectrum, doubling the volume of nanoparticles makes the absorbance band of Trenbolone almost fade away while at the same time, enhancing the LSPR band of silver nanoparticles at 411 nm. Therefore, the behavior of intensity of the Tren-specific absorbance band is inverse to the behavior of the LSPR of silver nanoparticles. This result is expected because increasing the concentration of silver nanoparticles means that more silver nanoparticles are now absorbing more light at the expense of Trenbolone. This observation was equally noted by^45^ who explored the impact of cyanide on the LSPR band intensity of AgNPs. The authors noted that the intensity of the SPR band reduced with an increasing amount of cyanide ion which, the author suggested was due to decreasing concentration of AgNPs. Fu et al.^44^ optimized the best ratio of silver nanoparticles to be applied in mercury detection. The authors noted that a ratio of 1:0.5 for AgNPs to Mercury gave the best results. However, for this work, a ratio of 1:1 was maintained since it helped in understanding the influence of the interaction of trenbolone with AgNPs without loss of signal information.

### Effect of Trenbolone concentrations on the AgNPs LSPR

**Figure 4:**
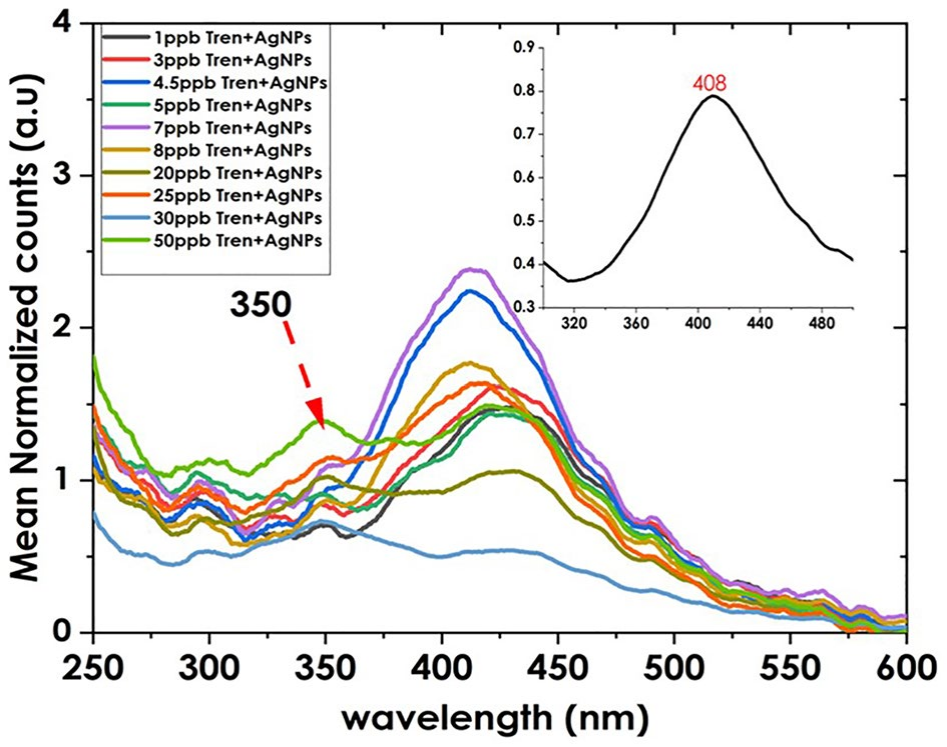
Effect of Trenbolone concentrations on LSPR behavior of Silver Nanoparticles. The nanofluid used was for same concentration (inset: spectra of AgNPs).

The impact of trenbolone on the localized surface plasmon band of silver nanoparticles was explored in this section as shown in Fig. 4. To have a clear view of the change in the LSPR band, the ratio of Tren: AgNPs was maintained at 1:1.

Several observations can be drawn from the UV–Vis characterization of trenbolone when mixed with silver nanoparticles. One impact that Trenbolone had on the AgNPs was the reduction in the intensity of the LSPR band. Caro et al.^46^ explained that one of the reasons is scattering/absorption competition since analytes can absorb or scatter light in the same wavelength range as the plasmon resonance of silver nanoparticles. Consequently, there is a possible reduction in the intensity since some of the incident light is absorbed or scattered by the analytes instead of interacting with the nanoparticles. Furthermore, analyte molecules may adsorb onto the nanoparticle surface and form a coating or layer, altering the optical properties and changing the absorption characteristics and intensity of the nanoparticles. For instance, Hajizadeh et al.^45^ attributed the reduction of intensity to the oxidation of silver nanoparticles due to dissolved oxygen from CN^-^ ions from cyanide and the subsequent formation of a complex mixture.

**Figure 5:**
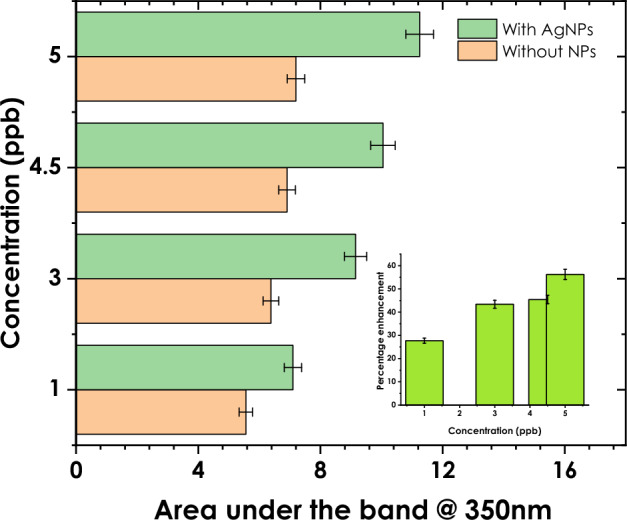
Signal enhancement for the first four concentrations (inset: percentage enhancement as a factor of concentration).

Silver nanoparticles are also known to exhibit a localized surface plasmon resonance (LSPR), which is responsible for their strong absorption and scattering of light. However, Trenbolone interacts with the surface of the nanoparticles and modifies the local electromagnetic field, thus altering the LSPR behavior^46^. The second observation is the signal enhancement of Trenbolone as can be witnessed in the band at 350 nm for the selected concentrations. The signal enhancement for the first four samples is shown in Fig. 5.

As shown in Fig. 5, there is a relative increase in the absorbance when trenbolone is mixed with silver nanoparticles. The signal enhancement given as a percentage of enhancement (inset in Fig. 5) ranges from 28% at 1 ppb to 56% at 5 ppb. The enhancement is attributed to the strong near-field electromagnetic amplifications that are induced by the optical excitation of silver nanoparticles^47^. Jans and Huo^48^ also notes that the changes in the localized surface Plasmon play a critical part in the signal enhancement.

**Figure 6:**
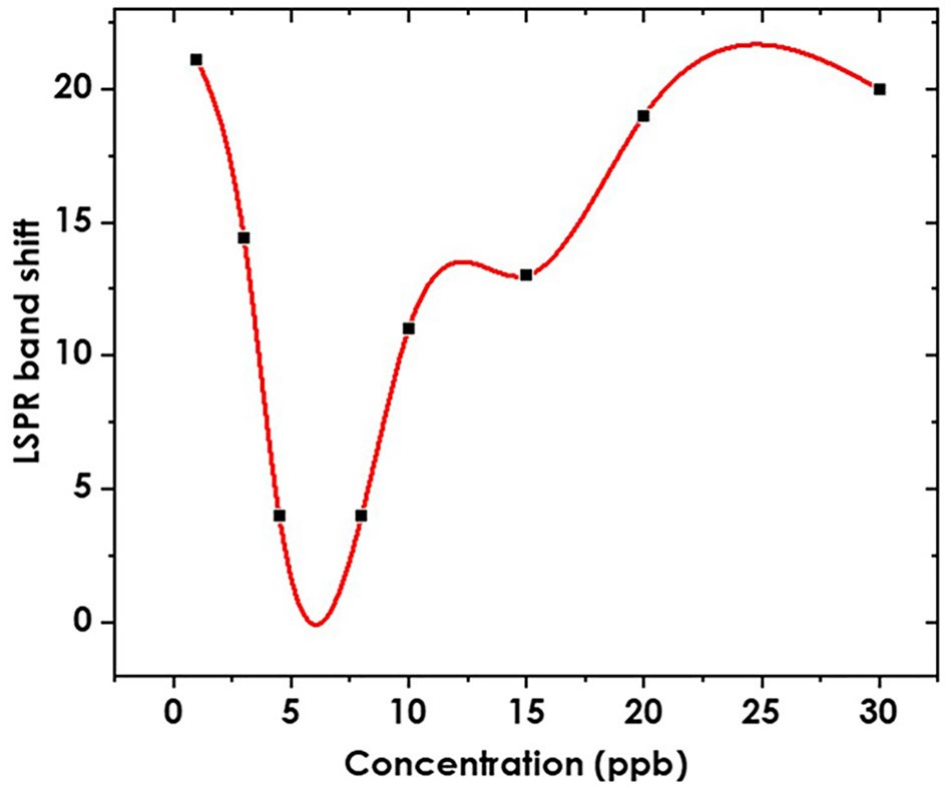
LSPR Red shift (nm) as a factor of concentration.

Other than the reduction in the intensity of the LSPR band of silver nanoparticles, a key observation used in plasmonic sensing is the shift in the LSPR. As shown in Fig. 6, there is a red shift in the plasmon band with changes in concentrations. The LSPR shift reduces and then increases exponentially with an increase in analyte concentration. At low analyte concentrations, the analyte molecules or particles interact with the metal nanoparticles individually, resulting in weak electromagnetic field coupling. It is this weak coupling that leads to a broadening of the LSPR band due to variations in the local refractive index surrounding the nanoparticles. As a result, the LSPR band appears broader and less defined^49^. However, at higher concentrations, there is a higher probability of analyte molecules or particles coming into proximity with each other and interacting with multiple nanoparticles simultaneously. This can lead to aggregation or clustering of the nanoparticles, resulting in enhanced electromagnetic field coupling^26^. The aggregated nanoparticles exhibit stronger interactions, which can narrow the LSPR band and make it more distinct^50^.

**Figure 7:**
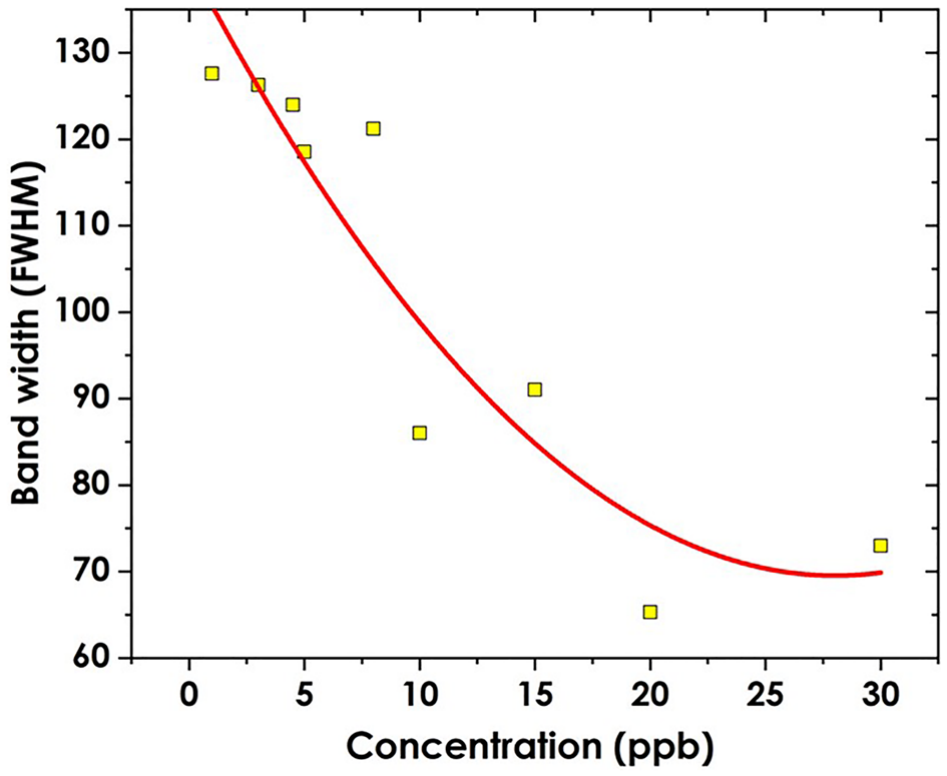
Band broadening of the AgNPs band as a function of concentration for Trenbolone Acetate.

The lack of linearity in the LSPR band shift reported in Fig. 6 as well as the band broadening effect given as FWHM Fig. 7 show that the interaction of nanoparticles and the target analyte is not uniform. Palani et al.^50^ notes that the variation in both intrinsic properties like geometry and the extrinsic properties like the environment results in inhomogeneity of the LSPR which eventually impacts the overall sensing response. The inhomogeneity can also explain why the LSPR band shift negatively correlates with concentrations at concentrations lower than 5 ppb as shown in Fig. 6.

Since LSPR is highly sensitive to the target analyte bound to the nano surface, the high refractive index of the AgNPs/Tren Ac complex is expected to amplify the LSPR shift after binding. This observation enables ultrasensitive detection of biomolecules. The results in Fig. 6 conform to the Drude model which states that there is an almost linear dependence of LSPR wavelength and the refractive index of the surrounding medium^24^, in this case, noted after 5 ppb.

### Effect of concentration on band broadening given as FWHM

The performance of any sensor technique can be evaluated using several parameters including sensitivity, detection limit, quality factor, and the figure of merit among others^51,52^. The Figure of merit is particularly important since it is directly proportional to the sensitivity and inversely proportional to the full width at half maximum of the transmission/ absorbance band as shown in Eq. (1). Similarly, the value of the signal-to-noise ratio can as well be explored from the FWHM.

3 $$FOM=\frac{S}{FWHM}$$

A reduction in the FWHM also results in increasing the quality factor of any sensor^53,54^. Since the ultimate goal of this research is to explore the feasibility of silver nanoparticles towards optical biosensing, it is necessary to explore the FWHM as a factor of analyte concentration. Figure 7 provides the trend of change in bandwidth (FWHM) for silver nanoparticles after mixing with Tren Ac.

**Figure 8:**
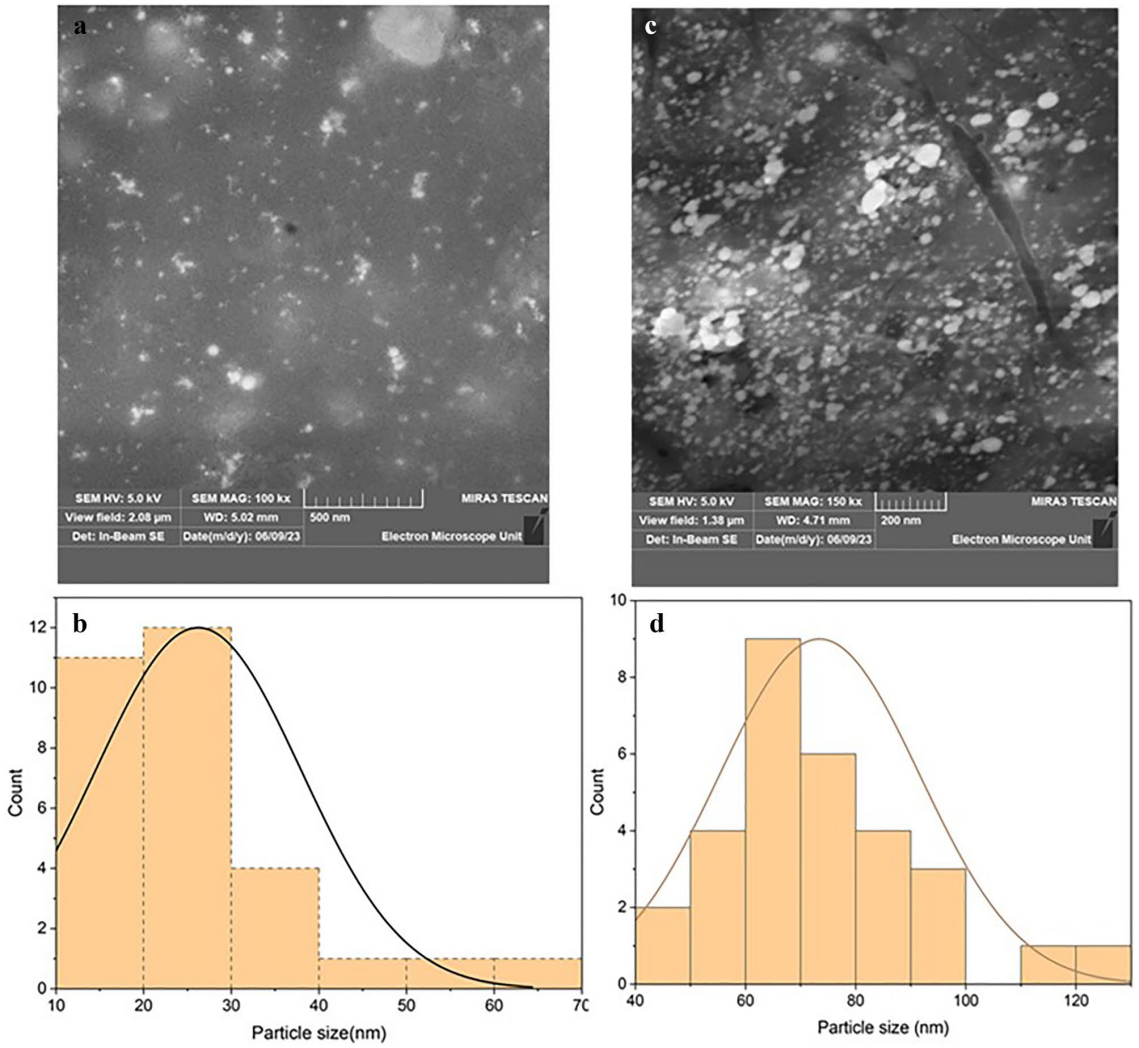
SEM Analysis of AgNPs (**a**) and its particle distribution in (**b**); Trenbolone/AgNPs complex, (**c**) and its particle distribution (**d**).

The band-broadening effect can be noted in this case. The broadening can be used to explain the possible interaction of silver nanoparticles with trenbolone at different concentrations^55^. The narrowing of the LSPR band can be attributed to the decrease in the plasmonic coupling of the nanoparticles^56^ due to the interaction with trenbolone acetate. At lower concentrations, the electric field due to incident light is less affected as the number of silver nanoparticles is higher than those of trenbolone acetate^57^. Therefore, the interparticle interaction between Tren Ac and silver nanoparticles increases with an increase in concentration which results in the narrowing of the LSPR^55^. When nanoparticles are mixed with analytes, the interaction between the analyte molecules and the nanoparticle surface can modify the electronic transitions or energy levels available for light absorption^58^. This interaction may lead to changes in the FWHM compared to the bare nanoparticles. Another possible reason for the narrowing of the band is increased particle agglomeration as the concentration of trenbolone increases; defined as an ensemble effect by^59,60^. As the concentration increases, the adsorbing layer also increases as more particles tend to agglomerate as depicted using a scanning electron microscope in Fig. 8.

Results in Fig. 8 indicate that the particle sizes increased when silver nanoparticles were mixed with Trenbolone. The average particle size for silver nanoparticles was 26 nm while the Tren/AgNPs complex gave an average particle size of 74 nm. The increase in particle size also suggests agglomeration and confirms the conclusions drawn in the FWHM which showed that there was band broadening in the Tren/AgNPs complex. This result can further be explored using energy-dispersive X-ray spectroscopy (EDS).

### EDS Spectra of AgNPs and AgNPs/Tren Ac Complex

**Figure 9:**
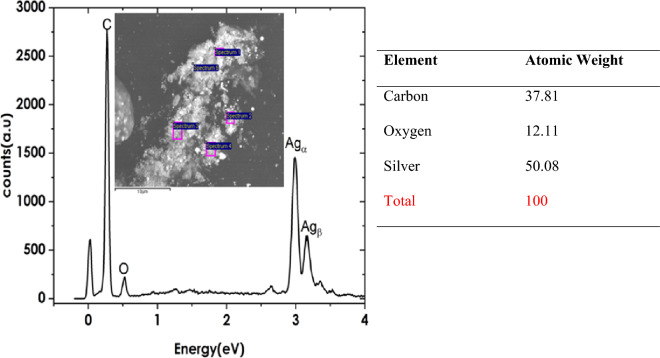
EDS Spectra of silver Nanoparticles (inset region of focus during spectral acquisition). On the right is a Table showing percentage concentration of atoms.

**Figure 10:**
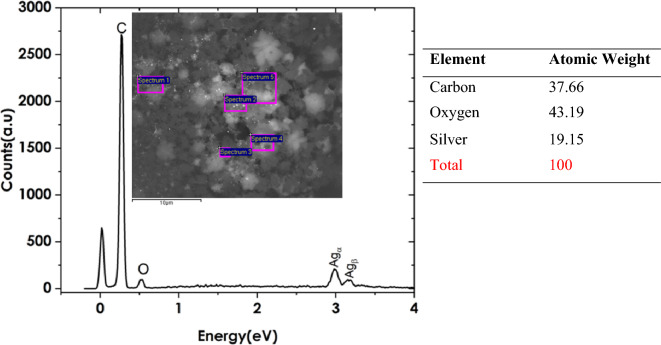
EDS Spectra of silver Nanoparticles/Trenbolone acetate Complex (inset region of focus during spectral acquisition). The table on the right shows the percentage concentration of the atoms.

To understand the coupling/agglomeration tendency of trenbolone acetate when mixed with silver nanoparticles, Energy Dispersive X-ray Spectroscopy (EDS) was carried out as shown in Figs. 9 and 10.

The EDS analysis of silver nanoparticles showed characteristic peaks at 3 eV and 3.2 eV as shown in Fig. 9. Oxygen was also evident at 0.52 eV. However, the presence of carbon is due to the carbon tape upon which the silver nanofluid is deposited for EDS analysis. Silver is the most abundant with a 50.08% concentration level. The oxygen is a result of water which was used as a solvent in the synthesis of silver nanoparticles. Figure 10 shows the changes in concentration of the elements when silver nanofluid was mixed with trenbolone acetate in the ratio of 1:1.

The three elements are still visible with the energies that were observed in Fig. 9. However, the concentration levels have changed especially for silver. After mixing, the intensity of silver reduces from 50.01 to 19.4% while that of oxygen increases from 12.11 to 43.19%. The percentages are taken as the averaged of five spectra taken from five different spots as indicated in the inset graphs. The reduction in the concentration of silver suggests that after mixing, most of the silver nanoparticles were obstructed from the oncoming radiations. The changes suggest that silver nanoparticles created a core–shell that was encapsulated by trenbolone acetate atoms^61^. Therefore, the oncoming radiations interacted more with the trenbolone acetate surface with less radiations passing through to the silver atom. The core–shell structure can also be explained by the increase in the concentration of oxygen. Trenbolone acetate is made of carbon, oxygen, and hydrogen. However, Hydrogen having a low atomic number, is not detectable using EDS. Since the concentration of oxygen also increased from 12.11 to 43.19%, we can decipher that most of the excited oxygen atoms were from trenbolone acetate as those from silver nanoparticles were surrounded by the adsorbed analyte atoms. The conclusion that can be drawn from the observation is that trenbolone molecules are agglomerated on the Ag NP surface in a core–shell-like structure.

## Computational results

The observed impact of Trenbolone on the LSPR of silver nanoparticles is likely to be a result of the strong electrochemical interactions. Therefore, it was necessary to investigate the Trenbolone acetate molecule’s HOMO and LOMO. Consequently, the structure of Trenbolone acetate was optimized at the B3LYP level of theory using the 6–311 basis set^62^ carried out using Gaussian 09. Using the same level of theory, simulated UV–Vis spectra were obtained as shown in Fig. 1. Frequency calculations were obtained on the optimized structure to achieve a true minimum. Thereafter, the orbital energies of HOMO and LUMO were calculated to get the quantum molecular descriptors^63^.

HOMO and LUMO which are described as frontier orbitals help to explain to explain the chemical reactivity of Trenbolone acetate and its subsequent molecular interaction. Figure 6 shows the molecular electrostatic potential (MEP) and the HOMO–LUMO orbitals of the optimized Trenbolone acetate structure. In the MEP, the reddish region shows the most active site (high electron density) of the molecule which suggests that oxygen atoms are the most active in interacting with silver nanoparticles. Silver ions which are positively charged will interact with negatively charged oxygen to form strong ionic compounds that are responsible for the noted changes in the LSPR^64^. Therefore, we can conclude that the redshift reported in Fig. 6 is a result of the growth of a shell of Ag_2_O around the particle^65^. The asymptotic behavior of the results also suggests that beyond 20 ppb, the shell thickness increases and reaches the saturation point where the plasmon peak remains at the same wavelength^66^. The refractive index of the created silver oxide tends to be much larger than that of Trenbolone acetate alone.

**Figure 11:**
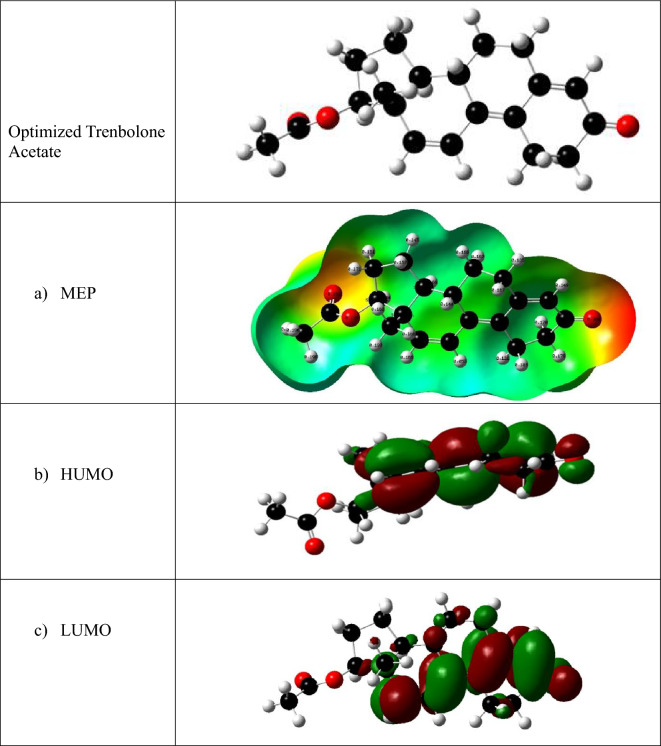
Molecular Electrostatic Potential (MEP) of the optimized structure of Trenbolone (the reddish regions indicate the most active sites of Trenbolone, (**b**) The Highest Occupied Molecular Orbital (HOMO), and (**c**) the Lowest Unoccupied Molecular Orbital (LUMO).

The Fig. 11 shows the optimized trenbolone acetate molecule, the molecular electrostatic potential describing charge distribution, and LUMO and HOMO for Trenbolone acetate.

The Fig. 11 shows that oxygen provides the electron-rich/dense region that will react with silver. Blue depicts the electron deficient, light blue provides the slightly electron deficient while yellow is slightly electron rich. Therefore, the carbons provide the least chance of the analyte's interaction with silver. For the optimized structural model, the HOMO is located along the most negative oxygen and the benzene ring. The presence of electrons along this oxygen and the active benzene ring provides a chance for chemical bonding/electronic transitions.

The derived energy of HOMO and LUMO orbitals and subsequently other chemical parameters that can explain the interaction of Trenbolone acetate and silver nanoparticles are summarized in Table 1. Chemical potential is often used to assess the evasion affinity of a molecule from equilibrium while chemical hardness is a property that quantifies the charge transfer and chemical reactivity of a molecule. Electronegativity and Electrophilicity index determine the ability of a molecule to attract electrons and electrophilic power of the molecule respectively. Therefore, while the DFT has provided insight into the opto-molecular behavior of trenbolone acetate, subsequent molecular dynamics would help to provide a theoretical evaluation of the interaction behavior and how it influences the LSPR behavior as noted experimentally. Nevertheless, the low band gap possibly shows that trenbolone acetate has a high chemical reactivity and as such, easily provides electrons for reaction as evidenced in the experimental results.

**Table 1:** The HOMO and LUMO energy, energy gap (eV), chemical potential (μ), chemical hardness (η), electronegativity (ξ), and electrophilicity (ω), (in eV) of Trenbolone Acetate.

E_HOMO_	The energy of the Highest Occupied Molecular Orbital	− 0.22365 eV
E_LUMO_	The energy of the Lowest Unoccupied Molecular Orbital	−0.08409 eV
E_gap_ = E_LUMO _− E_HOMO_	Energy gap	+ 0.13965 eV
µ = (E_LUMO_ + E_HOMO_)/2	Chemical potential	−0.15387 eV
ɳ = (E_LUMO _− E_HOMO_)/2	Chemical hardness	+ 0.069825 eV
χ = -(E_LUMO_ + E_HOMO_)/2	Electronegativity	+ 0.15387 eV
$\omega = \frac{{{\upchi }^{2} }}{2\eta }$	Electrophilicity index	+ 0.1696 eV

## Conclusion

This work has demonstrated the possibility of developing a simple sensing application of LSPR-active silver nanoparticles by detecting changes in the refractive index of the environment as depicted in the shifts and broadening behavior of the LSPR wavelength. The plasmon resonance wavelength shift is asymptotic to the small changes of the refractive index defined by the interaction of silver nanoparticles and trenbolone at different concentrations. Since LSPR sensing is often based on spectral peak shifts, the peak line widths also came in handy to explain the influence of trenbolone on the LSPR band of silver nanoparticles. The work has presented a label-free sensing approach by measuring the signal changes of the LSPR of silver nanoparticles in the presence of trenbolone acetate as the only target molecule. Unlike many other biological sensors that leverage labels to generate and amplify the target signal, the LSPR changes in this work were majorly based on the changes in refractive index and aggregation behavior as noted in changes in the particle sizes given by SEM.

Density functional theory helped to explore the opto-molecular properties of trenbolone acetate and subsequently, its interaction behavior with silver nanoparticles. The molecular electrostatic potential map, LUMO and HOMO show that the oxygen ions provide the most active sites for interaction with the nanoparticles and the formation of the Tren Ac/AgNPs complex. The coupling behavior allows the detection of minor changes in the LSPR band, a behavior that allows a label-free sensing approach. The change in the LSPR behavior could be attributed to the AgNP's phase transition and shell thickness and the aggregation of NPs. The presence of silver ion/Tren Ac mixture encapsulated the silver nanoparticles altering the plasmon behavior. The signal enhancement behavior of NPs and their impact on the localized surface plasmon can thus be extended in biosensing. The results show that localized surface plasmon resonance can be applicable in an anti-doping campaign which requires a sensitive, cost-effective, and label-free approach that leverages minimal sample volume.

## Data Availability

The datasets generated during and/or analyzed during the current study are available from the corresponding author upon reasonable request.
